# Understanding the importance of exposomics in everyday life: an interview with Emily Holden

**DOI:** 10.2144/fsoa-2020-0125

**Published:** 2020-11-09

**Authors:** Emily Holden

**Affiliations:** 1 The Guardian, 900 17th St NW, Suite 250, DC 20006, USA

## Please could you introduce yourself & provide a brief summary of your career to date?

I am the Washington, DC-based environment reporter for *The Guardian US*. I have previously covered environment agencies and the White House for Politico, E&E News and CQ Roll Call. I grew up in Baton Rouge, Louisiana and graduated from Louisiana State University (LA, USA).

## You have recently been awarded with the Endocrine Society Award for Excellence in Science & Medical Journalism based on your article ‘*Is modern life poisoning me? I took the tests to find out’* please could you provide an overview of this work?

This story was the launch piece for a Guardian series called Toxic America, where we explored how everyday chemical exposures affect Americans and why the regulatory system is so much more lenient here than in Europe and the UK. We wanted our readers to be able to relate to this very complex topic, so we decided a first-person piece would be best for explaining why an individual should care about the chemicals used in their cosmetics, furniture, food and water. I decided to seek testing of my own body and discovered that it is nearly impossible for the average American to do so. We eventually partnered with researchers Mount Sinai (NY, USA) and Oregon State University (OR, USA) to obtain the limited information that is available. Mount Sinai tested urine samples for a range of chemicals that are linked with various health problems. Oregon State provided me with a wristband that could detect chemicals I encountered in the environment that might not readily show up in a urine or blood test.

## What did the study results show?

Essentially, I have many of the chemicals in my body that you might expect for an American living in a city. What is more difficult to determine is whether the amounts and mixtures of the chemicals that I have absorbed might be dangerous to me. The urine tests showed 36 chemicals in my body including phthalates, which are used to make plastics flexible; and flame retardants, which are found in many home furnishings. They also revealed pesticides, phenols (likely also from plastics), polycyclic aromatic hydrocarbons from air pollution and cotinine from second-hand cigarette smoke.

The wristband detected 12 chemicals out of the 1530 it analyzed. Most were fragrances used in body care products and cleaning supplies. Some were phthalates, and one was a flame retardant. Phthalates were my major concern because they can mimic hormones and disrupt the endocrine system, and because they are easily avoidable if you reduce your plastic usage – which I had been motivated to do already. Unfortunately, there are few ways to measure childhood exposures, which can have a big impact on health. If I could have found one of my baby teeth, we would have tested that too. Alas, my parents could not locate it.

## How did you become involved in researching the science of exposomics?

In our first brainstorm for ideas on Toxic America, I suggested that we should test a group or Americans or even just one of our writers for exposures. I quickly realized how difficult that would be. I became even more fascinated with the field of study after long discussions with Robert Wright at Mount Sinai and Leonardo Trasande at the New York University (NY, USA). In climate science, we have many of the answers we need about what is happening and how to make it stop. In exposomics, the solutions are much blurrier. We still do not understand how the chemicals we absorb – almost all of which are unregulated in the US – affect us. For those that we do have good information on, we are largely ignoring that information.

## The science of our exosome in relation to endocrinopathies & oncology is an underrepresented field. Why do you believe that this area of research is growing so slowly?

I think Americans have been taught to believe that if something bad happens to their bodies, it is either inevitable or it is their fault. Our health system focuses on lifestyle and just ignores environment. That is baffling to me. I have never had a doctor ask me a single question about my home, my workspace or my possible childhood exposures (which were likely high because I grew up so near to petrochemical facilities). Patients do not yet know to ask doctors about these things. As they learn more about how the government is failing to protect them, I think they will demand better information.

## You state in the article that performing this study made you somewhat more conscious of your surroundings & what products you were using. Do you think that this had any negative psychosocial implications, as indicated by Paolo Vineis (Imperial College London, UK)?

As a reporter, particularly an environment and climate reporter in the US, I know a lot of things that are unsettling. I think that background has taught me to compartmentalize and keep some mental space from my work. That said, I am glad that I know so much more about my exposures because I can make very easy decisions that help to limit them. As long as I am doing the best I can, I feel good about that. I am sure, however, that many people would have trouble stomaching what they find. Knowledge allows us to make personal decisions to protect ourselves, but it also allows us to demand better of our leaders. That is worth any emotional toll it might take, in my opinion.

## As it has been a year since this story was published, what would you say was the biggest take home message & how has your lifestyle changed since doing this story?

Overall, I eat as many fresh fruits and vegetables as possible. I exercise, and I do everything I can to limit my stress. Every health professional I spoke with, said those are the three most important things to do to strengthen your body so it can handle the exposures of the modern world.

I also avoid plastics whenever possible, especially thin plastic packaging with hot food. I buy fewer things in general because many are hard to find without plastic of some sort. This is a lifestyle change I have made both to limit chemical exposures and to limit the harm, I take part in when I buy plastic – from its fossil-fuel-based production to its contamination of poorer countries and the oceans.

I filter my water with a filter that I know catches the major contaminants that are common in my specific water system. I am much more careful about anything I put on my body because we know the skin very easily absorbs chemicals. I use cleaning products with only naturally-derived ingredients, especially in my shower and tub. I avoid any synthetic fragrance, including in candles. I have a well-tested air filter in my bedroom and another in my kitchen. When I cook with the gas stove in my rented apartment, I use the back burner, open the window and turn on fans.

I have become a much wiser consumer when major purchases are necessary. We recently bought a new sofa and I insisted that it should not have flame retardants. It was surprisingly hard to find.

